# Emergence of *Brachyspira* species and strains: reinforcing the need for surveillance

**DOI:** 10.1186/s40813-015-0002-1

**Published:** 2015-06-12

**Authors:** David J. Hampson, Tom La, Nyree D. Phillips

**Affiliations:** School of Veterinary and Life Sciences, Murdoch University, Murdoch, WA 6112 Australia

**Keywords:** Brachyspira, Spirochaete, Swine dysentery, Species, Strains, Antimicrobial resistance, Virulence

## Abstract

This short review discusses the increasing complexity that has developed around the understanding of *Brachyspira* species that infect pigs, and their ability to cause disease. It describes the recognition of new weakly haemolytic *Brachyspira* species, and the growing appreciation that *Brachyspira pilosicoli* and some other weakly haemolytic species may be pathogenic in pigs. It discusses swine dysentery (SD) caused by the strongly haemolytic *Brachyspira hyodysenteriae*, particularly the cyclical nature of the disease whereby it can largely disappear as a clinical problem from a farm or region, and re-emerge years later. The review then describes the recent emergence of two newly described strongly haemolytic pathogenic species, “*Brachyspira suanatina*” and “*Brachyspira hampsonii*” both of which appear to have reservoirs in migratory waterbirds, and which may be transmitted to and between pigs. “*B. suanatina*” seems to be confined to Scandinavia, whereas “*B. hampsonii*” has been reported in North America and Europe, causes a disease indistinguishable from SD, and has required the development of new routine diagnostic tests. Besides the emergence of new species, strains of known *Brachyspira* species have emerged that vary in important biological properties, including antimicrobial susceptibility and virulence. Strains can be tracked locally and at the national and international levels by identifying them using multilocus sequence typing (MLST) and comparing them against sequence data for strains in the PubMLST databases. Using MLST in conjunction with data on antimicrobial susceptibility can form the basis for surveillance programs to track the movement of resistant clones. In addition some strains of *B. hyodysenteriae* have low virulence potential, and some of these have been found to lack the *B. hyodysenteriae* 36 kB plasmid or certain genes on the plasmid whose activity may be associated with colonization. Lack of the plasmid or the genes can be identified using PCR testing, and this information can be added to the MLST and resistance data to undertake detailed surveillance. Strains of low virulence are particularly important where they occur in high health status breeding herds without causing obvious disease: potentially they could be transmitted to production herds where they may colonize more effectively and cause disease under stressful commercial conditions.

## Introduction

Swine dysentery (SD) was first recorded in the mid-west of the USA in the 1920s but the aetiological agent was not discovered for another 50 years. Classical SD is seen mainly in grower and finisher pigs in which it manifests as an acute and severe mucohaemorrhagic colitis. On the other hand the disease may be mild and/or not clinically apparent in some herds, particularly where antimicrobial agents that may suppress the infection are used on a regular basis. SD has a worldwide distribution and is endemic in many countries where it can cause substantial financial losses through reduced and uneven growth rate, mortalities, costs of treatment and impediment to trade. It also may become a welfare issue where it is not effectively controlled.

In the early 1970s a strongly haemolytic anaerobic intestinal spirochaete named *Treponema hyodysenteriae* [1], but now known as *Brachyspira hyodysenteriae* [2], was shown to be the aetiological agent of SD. The spirochaete grows slowly and requires incubation for at least 3–5 days on specialized selective media in an anaerobic environment. Later in the 1970s a weakly haemolytic spirochaete that had been isolated from healthy pigs and did not cause disease was named *Brachyspira innocens* [3]*.* For diagnostic purposes it was important to be able to differentiate these two species, and this was done on the basis of their phenotypic properties, with the pathogenic *B. hyodysenteriae* being strongly haemolytic and indole positive and *B. innocens* being weakly haemolytic and indole negative.

This review gives a background to how this simple understanding of two species infecting pigs changed to a more complex one, and describes the emergence of both new *Brachyspira* species and of new strains, and their impact on diagnosis and control of disease caused by *Brachyspira* species in swine. Some details about changes to the genus and species names of these spirochaetes have been covered in an earlier review [4].

## Review

### Recognition of new weakly haemolytic *Brachyspira* species

From the late 1970s until the early 1990s, following examination of biochemical properties of intestinal spirochaete isolates it started to become evident that there was likely to be more than one weakly haemolytic *Brachyspira* species (WHBS) that colonized swine, and there was increasing awareness that sometimes WHBS could cause disease [5]. Due to the lack of simple molecular genetics techniques at that time investigations were based on examining phenotypic properties of isolates, and it was only with the application of multilocus enzyme electrophoresis and sequencing of the 16S rRNA gene in the early 1990s that it was shown that there were at least three more WHBS that colonized swine [6–8]. Of these, *Brachyspira pilosicoli* was clearly confirmed as being an enteric pathogen in pigs, causing a mild colitis and a diarrhoeal disease called porcine intestinal spirochaetosis [9, 10]. Interestingly this species also was shown to colonize and cause a similar disease in a number of species, most notably in chickens [11, 12] and human beings [13, 14]. Of the other two WHBS, *Brachyspira murdochii*, although generally considered a commensal, occasionally has been associated with mild colitis in swine [15–17]. The significance of *Brachyspira intermedia* in pigs is unclear [18, 19] – although it occurs commonly in adult chickens in which it is considered to be a pathogen [20].

### New diagnostic methods

The description of the pathogenic *B. pilosicoli* required the development of methods to be able to detect and differentiate it from other WHBS. Species-specific polymerase chain reactions (PCR) were developed for both *B. pilosicoli* and *B. hyodysenteriae* [21], even though the strong haemolysis of *B. hyodysenteriae* was still considered to be the gold standard for its identification. This new technology increased the speed of diagnosis, and it was further improved by development of duplex PCR systems [22], and later by the use of multiplex quantitative real-time PCR [23]. The techniques could be used on DNA extracted directly from faeces, although this procedure may be less sensitive than culture [24]. As a result of the availability of the new techniques, and because of the difficulty in isolating and identifying the anaerobic and fastidious *Brachyspira* species, by the mid-2000s a number of diagnostic laboratories had stopped using culture as part of the diagnostic protocol for detecting *Brachyspira* species in swine and simply relied on PCR techniques applied to faecal DNA.

### Cyclical changes in the prevalence of swine dysentery

Although SD is an endemic disease in many countries, within regions as well as on individual farms clinical disease has been observed to occur and later wane, with the latter probably resulting from enhanced diagnostic surveillance and implementation of control measures. For example, transmission of the disease between farms can be reduced by developing improved biosecurity measures, including quarantining of introduced stock [25]. Interestingly, it has been observed that the disease may re-emerge as a regional clinical problem in a cyclical fashion every five years or more. This phenomenon is not always explained, but one way that it can occur is through the movement of sub-clinically infected pigs from multiplier herds in apparently high health status production pyramids [26]. Other possibilities are the acquisition of strains from potential reservoirs such as migratory birds or feral animals [27, 28]; changes in strain properties so that the infection is amplified and transmitted more readily; reduction in herd immune status; or relaxation of routine diagnostic and control measures so that the disease gains the opportunity to spread. Low-level or subclinical colonization can exist on farms for many years, and then the disease can re-emerge when there are changes in management, including changes in diet [29, 30], co-infections with other enteric pathogens, or when other stressors occur. For example, stress induces release of norepinephrine into the gut, and this hormone has been shown to enhance *B. pilosicoli* growth and colonization [31]. The same is likely to apply to *B. hyodysenteriae*. A good example of a change in disease expression having occurred under different husbandry conditions was when an isolate from a pig in a high health status herd that had minimal antimicrobial usage and no clinical disease was shown to cause typical SD when it was used to experimentally infect pigs in a research facility [32]. If such subclinically infected pigs were transferred to a commercial piggery they could initiate clinical SD there. New and inexpensive screening methods such as serological ELISAs that can be applied routinely to large numbers of animals are needed to allow detection of these sorts of subclinical infections on farms [33].

In the USA clinical SD was common and widespread in the 1980s, but by the early 1990s it had become uncommon. This change was attributed to the introduction of medicated early weaning practices, the replacement of small family farrow-to-finish piggeries in the mid-West with new large multisite piggeries, often in non-traditional pig farming areas, introduction of all-in all-out pig flow, and better farm management. It also was suspected that the regular use of antimicrobial agents, and particularly carbadox, could have been suppressing disease. SD was no longer considered to be clinically important, and research and routine surveillance in North America was scaled down. Remarkably, and perhaps predictably given the history of this disease elsewhere, in the mid-2000s SD caused by *B. hyodysenteriae* re-emerged in the USA and Canada, and again started to become a common problem. This re-emergence has not been completely explained, but it followed the 2001 stop sale order on carbadox by Canada and the setting of zero tolerance for carbadox residues on US pork by Canada in 2007. In addition, and as previously mentioned, the movement of subclinically infected pigs may well have been a contributing factor to spread of the disease. A multilocus sequence typing (MLST) analysis of 59 *B. hyodysenteriae* isolates collected from farms in the USA post-2010 identified 13 sequence types (STs), including a predominant genotype (ST93), all of which were different to those from other countries [34]. Some of these STs showed genetic similarity to those of one or more of the 10 North American isolates from the 1970s−1990s that were analysed. These results suggest that these original strains may have persisted and been the source of the re-emergence of SD in North America, rather than completely different strains having been introduced from other sources [34].

### New strongly haemolytic species

In the early 2000s strongly haemolytic spirochaetes were isolated from feral ducks in Scandinavia [27]. Subsequently these spirochaetes, which formed the new proposed species “*Brachyspira suanatina*”, were shown to occur sporadically in pig in this region and to induce diarrhoea and changes consistent with mild SD in experimentally infected pigs [35]. To date this spirochaete has not been found outside the region. It is likely to occasionally spill over from feral and migratory ducks into susceptible pigs where it may cause a diarrhoeal disease resembling SD. This is most likely to occur in farms where pigs are kept outside, or where lagoon water contaminated with aquatic bird faeces is recycled to clean pig houses.

Later, around 2007, outbreaks of SD started to occur in Canada and the USA from which *B. hyodysenteriae* could not be detected or identified by PCR. Culturing of samples eventually identified the presence of another apparently novel strongly haemolytic species that was proposed as “*Brachyspira hampsonii*” [36]. Two main clades were identified and both of these were shown to be able to induce a disease indistinguishable from SD in experimentally infected pigs [37–39]. Subsequently this species was detected in lesser snow geese in arctic Canada [40], and then in over-wintering graylag geese and mallards in a nature reserve in Spain [41], as well as in pigs in Belgium and Germany (clade I) [42, 43]. A strongly haemolytic isolate designated P280/1 that had been recovered from a pig in the UK in the 1980s and had been thought to be a new species [44] also was shown to be closely related to “*B. hampsonii”* [36]. It seems likely that this emerging species has reservoirs in migratory water birds, and that the spirochaete may be transmitted from them to pigs to cause a disease that is indistinguishable from SD caused by *B. hyodysenteriae*. Like “*B. suanatina*” this new species was not detected by the PCR techniques that were in current use. The widespread distribution of this new species makes it more significant for porcine health than “*B. suanatina*”, although it should be remembered that surveillance for these and potentially other new pathogenic *Brachyspira* species is not routinely carried out in many regions of the world. Hence there is a very incomplete understanding of the occurrence and distribution of *Brachyspira* species at the global level, and more surveillance is required. As another example of this, a strongly haemolytic *Brachyspira* isolate that was distinct from *B. hyodysenteriae*, “*B. suanatina*” and “*B. hampsonii*” was isolated from an Australian pig herd with suspected SD [45]. Following treatment of the herd for SD this strain has not subsequently been isolated, nor has it been found in routine diagnostic submissions from other herds in the region. Hence the appearance of this atypical strain seems to have been a rare event, and one that easily could be overlooked.

It is interesting that these “emerging” pathogenic species have all been strongly haemolytic, and this correlation is consistent with the haemolytic activity contributing to virulence [38]. In some ways it was fortunate that these new species were strongly haemolytic, otherwise they may have taken much longer to identify. This series of events emphasizes the need to use both culture to obtain isolates and PCR methods for diagnosing *Brachyspira* infections, and to keep updating molecular diagnostic techniques. PCR methods based on the NADH oxidase (*nox*) gene are now available for “*B. hampsonii*” and “*B. suanatina*” [46, 47], and new rapid identification methods based on Matrix-assisted laser desorption ionization time-of-flight mass spectrometry (MALDI-TOF MS) are being implemented for identification of isolates of *Brachyspira* species [48, 49].

A number of these newly described weakly and strongly haemolytic *Brachyspira* species infecting pigs and other species still have “candidate” species status. It is important that additional phenotypic and genetic characterization is undertaken on members of these proposed species so that they can be more clearly identified and their names officially validated.

### New strains

Analysis of 16S rRNA gene sequences of the *Brachyspira* species has shown that they are all relatively closely related. Nevertheless all the species also show considerable strain diversity, and these strains may vary in their biological properties. Extensive genetic rearrangements have been identified within and between the species, with sequence drift also generating genetic diversity [50]. At a farm level “microevolution” of *B. hyodysenteriae* strains involving small genetic changes has been recorded over relatively short periods of time [51, 52]. Besides genetic rearrangements, novel genetic information may be acquired from other *Brachyspira* species or strains through the activity of prophage-like gene transfer agents that are present in the genome of different *Brachyspira* species [53, 54]. In addition, horizontal gene transfer via bacteriophages with broad trophism is likely to be an important force in the evolution of the *Brachyspira* species [55]. This knowledge has clear implications for control, as new strains, clonal groups and even new *Brachyspira* species are likely to emerge over time. One consequence of this emergence is that phenotypic properties that have been used to identify species may become less reliable than originally thought. For example, in the mid-1990s atypical indole-negative strains of *B. hyodysenteriae* were identified in Europe [56, 57], and recently atypical weakly haemolytic strains of *B. hyodysenteriae* that appear to have reduced virulence in pigs have been reported in Europe [58], and also detected in Australian pigs (Phillips ND, La T, Hampson DJ 2015. Unpublished data).

Strain variation has been studied in most detail in *B. hyodysenteriae,* using a variety of phenotypic and molecular methods. In recent years MLST has been used to identify new clonal groups of *B. hyodysenteriae* [59–61], and these can be tracked nationally and internationally by comparing their STs in the PubMLST database [62]. Within clonal groups a reduction in tiamulin susceptibility of isolates over time has been observed [63], and this emphasizes the power of combining MLST and resistance data for monitoring the development and spread of resistant strains. This capacity is particularly important as the emergence of resistant and multi-resistant *B. hyodysenteriae* strains can seriously compromise disease control [64, 65].

It has been known since the 1980s that different strains of *B. hyodysenteriae* vary in their pathogenic potential, with a minority being weakly virulent or avirulent [66–68]. Such strains, particularly if present in breeding herds, could cause a serious industry problem as the pigs may appear healthy but test positive for a major pathogen. Such a result can cause substantial disruptions to trade, even though the isolates may not be highly problematic from a clinical perspective. Alternatively such strains may not cause disease on the farm of origin, but perhaps cause disease when transferred to commercial herds [32]. Although there may be various reasons for apparent reduced virulence, including a lack of strong haemolytic activity by some strains [58], recently it has been demonstrated that *B. hyodysenteriae* strains that either lack the ~36 kB plasmid [69] or certain genes on the plasmid have low pathogenic potential [70, 71]. These strains, including the type strain B78^T^, appear to have a reduced ability to colonize, although they are capable of causing typical SD lesions if they do establish themselves sufficiently in individual pigs [67]. Lack of the plasmid or specific plasmid genes can be identified using PCR testing on isolates, and this information can be used to help identify individual isolates that are predicted to be less able to cause disease. The unusual *B. hyodysenteriae* strains that have weak haemolysis also may have reduced virulence, although further work is required to confirm this. Overall, a surveillance system that included information on clonal origin, antimicrobial resistance profile and virulence potential of isolates would be very valuable to help monitor and control the spread of SD at the local, national and international levels. Similar systems are required for other pathogenic *Brachyspira* species.

## Conclusions

Routine surveillance at local, national and international levels is required to monitor *Brachyspira* species infections in pigs, but also carriage in other species which may act as reservoirs of infection (particularly migratory water birds). Surveillance of the latter has the added benefit of increased preparedness should a new *Brachyspira* species spill over into pigs, as appears to have been the recent case with “*B. suanatina*” and possibly with “*B. hampsonii*”.

The various species in the genus *Brachyspira* have different population structures, but all are genetically quite plastic. Consequently existing non-pathogenic species may acquire determinants from other species, especially through the activity of gene transfer agents and bacteriophages, and become more virulent. Similarly new strains of the species routinely develop, and it is important to monitor their presence and potential spread.

Diagnostic methodology needs to be reviewed regularly, but should include both culture and molecular techniques. The cultural techniques have the benefit of providing isolates of the species that can be used to undertake molecular epidemiology studies, and assess antimicrobial susceptibility and virulence potential. Knowledge of these attributes and the spread of clonal groups is very important for implementing programs to control disease caused by *Brachyspira* species.
